# Defining the esports bettor: evidence from an online panel survey of emerging adults

**DOI:** 10.1080/14459795.2020.1826559

**Published:** 2020-10-06

**Authors:** Heather Wardle, Elena Petrovskaya, David Zendle

**Affiliations:** a School of Social and Political Sciences, University of Glasgow, Glasgow, UK; b Computer Science, University of York, York, UK

**Keywords:** Gambling, betting, esports, loot boxes, video games, young adults, survey

## Abstract

Competitive video gaming (esports) is a growing multi-national, billion-dollar industry. Esports cultures replicate traditional sports cultures, involving elite athletes, teams, league sponsorships, large viewing audiences, high profile leagues and championships, and opportunities to bet on outcomes. However, little is known about people who bet on esports, it is generally considered a niche practice. Using data from the Emerging Adults Gambling Study, a non-probability survey of 3549 people aged 16–24 living in Great Britain, the profile of esports bettors was compared with those who bet on other sports and non-gamblers. Those who bet on esports were more likely to be male, to be from nonwhite ethnic groups, to be heavily involved in playing digital games themselves, and to have higher rates of gambling involvement and problem gambling. Multivariate analysis showed a strong relationship between engaging in gambling-like practices within digital games and esports betting (for example, the purchase of loot boxes for money, or betting skins on external websites). Frequency of playing digital games was not associated with esports betting, suggesting it is not how often someone engages with digital games that is correlated with esports betting, but rather the different type of practices they undertake when playing video games.

## Introduction

The practice of engaging with competitive video game play as if it were a traditional sport is known as esports. In recent years, this practice has expanded rapidly: one estimate holds that the global esports market will grow to 495 million unique viewers in 2020 alone (Newzoo, 2020). Due to the nature of the way esports is presented and consumed, the rising esports industry brings with it in tandem an emerging large-scale betting market. Multiple papers suggest that the esports betting market can be estimated to be valued at approximately 5 USD billion in 2016, rising to over 12 USD billion by 2020 (Abarbanel et al., 2016; Macey & Hamari, 2018; Sweeney et al., 2019), and there is evidence to point to the prevalence of esports betting being widespread yet little is known about those who bet on esports (Gainsbury et al., 2017; Gambling Commission, 2017; Macey & Hamari, 2018).

Esports culture has developed in a manner that mirrors traditional sporting culture. A cadre of elite esports athletes have emerged who enjoy celebrity status and large earnings in a similar fashion to elite competitors in traditional sports. One esports player earned more than 3 USD million from tournament play in 2019 alone (Jenkins, 2020). Esports teams now have mascots and sponsorship deals – esports organization 100 Thieves are sponsored, for example, by the mainstream burrito giant Chipotle (Fitch, 2020). A host of secondary industries are associated with esports play. Esports commentators and coaches are thought to now regularly make a full-time living from their work (McKeever, 2020); multiple storefronts and merchandising companies exist that are dedicated to selling esports-related goods.

As with traditional sports, thousands of enthusiasts regularly travel internationally to attend gameplay tournaments of varying prestige. In 2019, *The International*, the highest status tournament within the Dota 2 community, was held in China and attracted around 60,000 spectators, around half of which were international fans. Unlike traditional sports, esports tournaments are more accessible to the average viewer: every game of most tournaments is broadcast live online. The value of such a medium is not going unnoticed – advertisers, including betting operators, are reported to pay large sums for their products to be associated with these tournaments (Schultz, 2017).

Arguably, viewing esports also facilitates a different kind of experience to that of other activities and traditional sports. Dubbed ‘participatory spectatorship,’ watching an esports match provides entertainment but also a socially engaging element of viewership (Georgen, 2015) as most live-streaming platforms provide an opportunity for real-time exchange between spectators. It is common for viewers to communicate their thoughts about a game on Twitter, or to connect with friends through the game communication software Discord alongside watching.

A unique feature of esports is that it is incredibly data-rich; remarkable volumes of data are outputted from every game (Block et al., 2018). The potential of such data has already been spotted by research groups and commercial organizations for advanced player tracking (Katona et al., 2019) and performance analysis (Schubert et al., 2016). This has potentially important implications for the development of esports betting, which rely on data to generate, regulate, and market products through a variety of media to consumers.

With the consolidation of esports as a professional and valid enterprise, it is unsurprising that a large-scale esports betting industry is starting to emerge, though there are few statements in the literature regarding esports betting that researchers can be confident about.

Some estimates of the prevalence of esports betting suggest that it may be widespread. The UK Gambling Commission estimated that 8.5% of adults have bet on esports; with 58% of esports bettors being men and 42% being women (Gambling Commission, 2017). Gainsbury et al. (2017), surveyed 501 Australian sports bettors, 160 of whom bet on esports. They found that esports bettors participated in significantly more forms of gambling, gambled more frequently, and had higher problem gambling scores. Macey and Hamari (2018) reported similar results from a self-selected survey of 582 esports spectators, finding that esports betting was common alongside other types of gambling, and over 50% of the sample experienced ‘problematic and potentially problematic gambling.’ Despite this, Greer et al. (2019) concluded that ‘reliable data on the prevalence, characteristics, and gambling behaviours of esports bettors are hard to obtain, considering that relatively few studies have been conducted’ and noted that use of non-probability sampling and sampling from self-interested groups (sports bettors or esports fans) has attendant issues of generalizability.

Understanding the characteristics of esports bettors is particularly important given the unique links between esports and video games, and its (relatively) recent emergence as an activity on which people can bet. Those betting on other sports are often strongly affiliated to specific sports through ties of fandom, familiarity, and family and peer networks. It would be surprising if esports bettors were not similarly affiliated to the products on which they bet. Thus, understanding the profile of esports bettors also requires attention to broader esports fandom as well as personal gaming practices. As noted in a report produced by the UK Gambling Commission, connections between esports and video games may mean that audiences are younger and maybe unusual in their composition (Gambling Commission, 2017). In terms of identifying new trends and cohorts interested in gambling, consideration of younger adults’ engagement with esports may be particularly insightful.

Esports bettors may also vary in terms of their engagement with specific video game practices. For example, they may be more likely to engage in gambling-like practices within video games, such as the purchase of loot boxes, or with gambling practices that surround video games, such as the betting and trading of skins. Research has suggested that loot boxes share key similarities with gambling, and their use has been linked to problem gambling (Brooks & Clark, 2019; Drummond & Sauer, 2018; Li et al., 2019; Zendle et al., 2020). Skins are in-game items which afford no in-game advantage and are purely cosmetic in nature. In skin gambling, these decorative in-game items are used as stakes in external games of chance, with their value acting as a proxy for fiat currency in a similar fashion to the operation of gambling chips in other activities. Situating esports betting within broader gaming-related consumption is therefore important (Macey et al., 2020). Few studies have looked at this, and even fewer have examined this for young adults specifically. Our understanding of who esports bettors remains nascent.

### Aims and objectives

The aims of this study were to conduct exploratory data analyses to examine the profile of esports bettors aged 16–24 recruited as part of a larger online survey of Emerging Adults (Wardle, 2020). Exploratory analyses sought to examine the demographic, socio-economic, gaming and gambling profiles of esports bettors. To ensure that any statistical differences observed represented the unique profile of esports bettors specifically rather than of gamblers generally, their profile was compared with that of sports bettors (deemed to be the closest comparator group to esports bettors), other gamblers and of non-gamblers. Multi-variate analyses also examined the extent to which engagement in digital games and gambling-like mechanics within them was associated with esports betting.

## Methods

The Emerging Adult’s Gambling Survey collected data from 3549 16–24 year olds. Participants were drawn from YouGov’s online panel of over one million people living in Britain and were eligible if they were aged between 16 and 24, living in Britain and had not taken part in any other YouGov study on gambling in the past year. E-Mail invites to participate were sent by YouGov to a random selection of their panel members, stratified by region. This e-mail asked them to take part in a survey, without advertising its content, and asked participants to click through to the bespoke study. The first page of the bespoke survey described the project and obtained consent; 93% of people who accessed this page went on to complete the survey. Data were collected between June and August 2019. The survey asked about gambling, gaming, social media, and health-related behaviors. Full detail on questionnaire development and quality assurance measures (including seriousness checks) are given in (Wardle, 2020).

Ethical approval for the study was granted by the London School of Hygiene and Tropical Medicine’s Research Ethics Committee (ref: 15960).

### Measures

Participants reported whether they had played any form of digital, computer or video game in the past year, and if so, how often. Those who did were asked how often they engaged in the following activities when playing video games: buying loot boxes with their own money; buying loot boxes with in-game currency; betting skins privately with other players/friends; betting skins in external websites. Answer options ranged on a four-point scale from very often to never.

Participants reported whether they have ever gambled, and if so how often they gambled, on a range of 18 different gambling activities legally available in Great Britain. This included whether they had bet on esports. Esports bettors were defined as anyone who reported betting on esports in the past year. Based on the activities reported, the following mutually exclusive groups were derived: whether they had bet on esports in the past year (irrespective of whether they had bet/gambled on other things); whether they had bet on other sports (excluding those who reported betting on esports) in the past year; whether they had gambled on other activities (excluding esports and sports betting) in the past year or whether they were non-gamblers. Problem gambling was measured using the Problem Gambling Severity Index (PGSI), a validated tool for the identification of gambling problems (Ferris & Wynne, 2001). The PGSI score ranges from 0 to 27; a score of 0 indicated non-problem gambling or non-gambling; 1–2 is low-risk gambling; 3–7 is moderate risk gambling, and a score of 8 or more is indicative of problem gambling.

Impulsivity was measured using a shortened form of the Eysenck Impulsivity Scale which is validated for use among adolescents (Eysenck & Eysenck, 1977; Wills et al., 1998). Participants were asked to respond on a five-point scale how true seven different statements about impulsivity are for them. Impulsivity scores are computed as the average of the seven questions [mean = 2 · 6], similar to other published reports among young people] (Auger et al., 2010).

#### Other measures

Ethnicity was self-reported using the harmonized ONS ethnicity question. Because of low base sizes, responses were grouped into White/White British, Asian/Asian British, Black/Black British, and Other. Age was captured in single age years. Local area-level deprivation was measured using the respective English, Scottish and Welsh Indices of Multiple Deprivation (IMD) scores matched at the ‘Output Area’ and quintiled for analysis. Respondents were asked to report their current economic activity. From this those who were students, either full time or part time, were identified, as were those not in education, employment, or training.

### Statistical analysis

Unadjusted bi-variate associations between gambler type and socio-demographics, economic status, impulsivity, gaming, and, where appropriate, gambling behaviors were examined. All analyses used the complex survey function in SPSS v20 to adjust for the weighted stratified survey design. These complex survey modules produce a Walds F-test as the default test of significance (Rao & Scott, 1984). For bivariate analyses, this assesses the extent to which the independent variable (gambler type, for example) varies by the dependent variables (age or problem gambling status, for example) and is the test on which all p-values presented in Table 1 are based. Two statistical tests were run, one to examine the extent to which dependent variable varied across all four categories of gambler (esports betting; sports betting; other gambling; no gambling) and another to assess if the dependent variable varied between esports betting and sports betting.

Multivariable binary logistic regression analysis was run in Stata v15, with past year esports betting entered as the dependent variable (Table 2). This also used the complex survey command to adjust for weighting and stratification. Because of the relatively sparse number of esports bettors (n = 103), regression models had to be conducted with care, limiting the number of dependent variables entered. For this reason, the regression model focused on the extent to which other gaming behaviors were associated with esports betting, with age, sex, and economic status as controls.

Diagnostic checks on multi-collinearity were conducted by calculating the variance inflation factors (VIF) of all independent variables, all had VIF values of less than 2 indicating they were not too closely correlated (Mansfield & Helms, 1982). The exceptions were betting skins privately and betting skins on external websites, which had VIF values of 2.09 and 2.05, respectively, as these values were close to the threshold of 2, both were included. All estimates were weighted to match the age, sex, and regional profile of 16 to 24 year olds living in Great Britain. True (unweighted) bases are presented.

## Results

**Figure 1:**
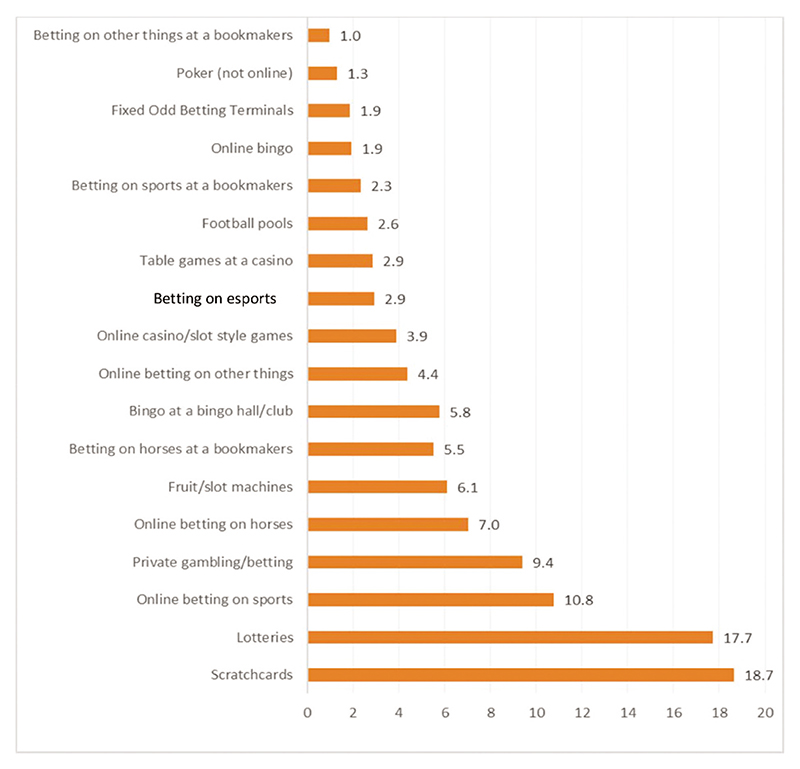
Past year participation in gambling activities.

Among young adults aged 16–24, 2.9% had bet on esports in the past year, making it as prevalent within our sample as playing table games in a casino, betting on sports in bookmakers or betting on Fixed Odd Betting Terminals in bookmakers (see Figure 1).

Compared with non-gamblers, esports bettors were more likely to be male (74% vs 49%), older (78% aged 20–24 vs 63% for non-gamblers), to be from nonwhite/white British ethnic groups (26% vs 17%); living in more deprived areas (33% vs 20%), to be a student (59% vs 42%) and to have higher impulsivity scores (mean scores 3.1 vs 2.1). They were less likely to be economically inactive (10% vs 15%). However, the socio-demographic/economic profile of esports bettors was similar to that of other sports bettors, the only difference being that esports bettors were even more likely to be male (74% vs 62%) and from nonwhite groups (26% vs 8%).

Esports bettors were much more likely to play digital games than both non-gamblers and also other sports bettors: 61% of esports bettors played digital games more than once a week compared with 43% for sports bettors and 38% for non-gamblers. Esports bettors were also more likely to have engaged in the purchase of loot boxes (either with their own money or in-game currency) or to have bet skins. The difference was most stark for betting skins on external websites: 58% of esports bettors did this compared with 7% for sports bettors and 2% for non-gamblers.

Compared with sports bettors and other gamblers, esports bettors were also highly engaged in gambling generally: 39% gambled more than once a week; 50% gambled on five or more different activities in the past year whilst 53% had a PGSI score of 8 or more, indicative of problem gambling. Among other sports bettors, equivalent estimates were 10%; 24% and 9%, whilst among other types of gambler, 3% gambled more than once a week, 1% gambled on five or more activities and 3% had a PSGI score of 8 or more.

Multivariate regression models looked at the association between engagement in certain digital gaming practices and esports betting. The odds ratio of being an esports bettor were higher among those who had purchased loot boxes with their own money fairly often/often than those who had not (10.40; 95% CI: 4.08–26.80); odds were also higher among those who had bet skins privately or on external websites. Notably, the odds of being an esports bettor did not vary based on frequency of playing digital games or among those who bought loot boxes using in-game currency.

## Discussion

Until recently, esports betting was considered a niche or periphery gambling activity: the preserve of those very engaged in gaming cultures. Whilst our study supports this view, the prevalence of esports betting among those aged 16–24 needs to be considered in context. For this age group, gambling on activities other than the National Lottery or scratchcards tends to be something that less than 10% of people engage in. That 2.9% of young people, or 6.4% of past year gamblers within our sample, engaged in esports betting makes this comparatively popular among those who gamble – as popular as visiting casinos, bookmakers, or playing fixed odds betting terminals.

Furthermore, this data was collected in July/August 2019, prior to the Covid-19 pandemic that precipitated unprecedented changes in behaviors, including the cancellation of major sporting events. Many industry commentators have viewed this as a catalytic moment for esports, where they *could* become a more mainstream betting activity, not being subject to the same restrictions as live sports. Monitoring this change is important as is understanding the changing demographic of who bets on esports. Exploratory analyses in this paper provide useful insight against which future changes may be assessed.

Consistent with previous studies, young adults who were esports bettors were more likely to be men, to be from nonwhite ethnic groups, to be heavily engaged in gaming and also heavily engaged in gambling (Gainsbury et al., 2017; Gambling Commission, 2017; Macey & Hamari, 2018). The prevalence of problem gambling among esports bettors was particularly notable and especially high when compared with bettors on other sports events (who arguably may be the closest comparator group to esports bettors). Whilst the frequency of playing digital games was associated with esports betting in unadjusted analyses, in multi-variate analyses it was not. When it came to gaming practices, the most strongly correlated practices were not how often you engaged in digital games but rather the actions you take when you play them: notably paying money to open lootboxes, betting skins privately with friends/other gamers and betting skins on external websites. There is a high degree of concordance between engaging in gambling-like practices within digital games (and without in the case of external betting of skins) and betting on esports.

Whilst some of this association may reflect that people who bet on esports may simply be those very interested in gambling-like mechanics in all their guises (and our analyses suggest this to be the case), it is also plausible that some of this relationship may be causal, with engagement in esports betting leading to engagement in gambling-like practices in digital games or vice versa. Longitudinal data are needed to assess this in greater detail.

There are a number of limitations associated with this study. As an online panel, YouGov also has issues of generalizability, though with respect to young people Wardle (2020) has argued that the YouGov panel has better sample coverage than household-based probability methods, which routinely exclude certain groups like students. This study arguably represents an advance on its predecessors, which have tended used self-selected samples from online platforms like Reddit or Amazon’s Mechanical Turk, both of which present specific limitations when it comes to sample coverage (Amaya et al., 2019; Mishra & Carleton, 2017). Whilst this study included data about gambling-like practices associated with video games, it did not collect data on esports fandom and associated practices, meaning we cannot fully situate our results within fuller gaming and esports repertoires. Furthermore, the relatively small number of esports bettors in the sample precluded detailed analyses of concordant gambling practices. Our finding that esports bettors tended to be highly engaged gamblers may be an artifact of sample size, as we had insufficient numbers to look at different types of esport bettors and simply designated anyone who had bet on esports in the past year an esports bettor irrespective of their other gambling engagement. This may also influence the associations observed with PGSI scores and problem gambling status, as esports bettors were highly engaged gamblers which may confound this association (LaPlante et al., 2014). As with all studies, especially non-probability samples, further replication is needed and these findings should be treated as preliminary.

When it comes to the relationship between esports betting and broader gaming practices, our data suggest, that among young adults, it is not how often you engage in digital games but rather the broader gambling-like practices that you engage in that are most highly associated with esports betting. To better understand these relationships, research needs to consider the fuller context of gaming and gambling behaviors – perhaps because of subject specialisms, research to date (our own included) has tended to focus on one and not the other – yet this preliminary data suggest that to understand esports betting, one needs to contextualise this within peoples broader gaming and gambling repertoires, which should include both sporting and esports fandom. This is especially the case for young adults, who have heightened interest in gaming cultures and for whom concerns about the ‘normalization’ of gambling have been raised. Our analysis suggests that young adults who are esports bettors are highly engaged gamblers, and highly engaged in gambling-like practices within digital games. Both of these features are, in turn, highly associated with increased levels of harm from gambling. Given that the profile of esports bettors may even be changing in response to Covid-19, these preliminary data suggest that young adults who are esports bettors could be considered a group highly vulnerable to potential gambling harms.

## Figures and Tables

**Table 1:** Profile of past year esports bettors^a,b^

	Esports bettors (n = 104)	Bettors on other sports/events (n = 584)	Other gamblers (n = 829)	Non-gamblers (n = 2033)	Total
	%	%	%	%	%
**Sex**					
Male^(a: p<0.01; b: p<0.05)^	74	62	48	49	51
Female	26	38	52	51	49
**Age group**					
16-18^(a: P<0.01; b; P=0.39)^	22	19	35	38	33
19-21	40	34	29	31	31
22-24	38	47	37	32	36
**Ethnic group**					
White/White British ^(a: p<0.01; b: p<0.01)^	74	92	89	83	86
Mixed	6	3	3	4	4
Asian	11	3	5	10	8
Black	8	1	2	2	2
Other	1	1	0	1	1
**Index of Area Deprivation Quintile**					
Least deprived	10	22	20	20	20
2^nd^	18	21	18	20	19
3^rd^	15	19	17	21	20
4^th^	24	16	21	20	20
Most deprived ^(a: p<0.05; b: p=0.11)^	33	22	24	20	21
**Economic activity**					
Not in education, employment or training ^(a: p<0.01; b: p=0.79)^	10	11	9	15	13
Full time or part time student ^(a: p<0.01; b: p=0.75)^	59	58	48	42	46
**Frequency of playing digital games**					
More than once a week ^(a: p<0.01; b: p<0.01)^	61	43	43	38	41
About once a week	20	12	11	9	10
About once a fortnight	8	6	9	8	8
About once a month	4	9	9	8	8
A few times a year	-	8	8	9	8
Not in the past 12 months	6	21	20	29	25
**Use of in-game items in past year**					
Had purchased loot boxes with own money ^(a: p<0.00; b: p<0.00)^	72	19	11	8	12
Had purchased loot boxes with in-game items ^(a: p<0.01; b: p<0.01)^	84	46	42	32	38
Had bet skins on external websites ^(a: p<0.01; b: p<0.01)^	58	7	4	2	5
Had bet skins privately ^(a: p<0.01; b: p<0.01)^	57	9	5	2	6
**Frequency of buying loot boxes with own money**					
Very often/fairly often ^(a: p<0.01; b: p<0.01)^	34	3	1	1	2
Occasionally	38	16	10	7	10
Never	28	81	89	92	88
**Frequency of buying loot boxes with in-game items**					
Very often/fairly often ^(a: p<0.01; b: p<0.01)^	44	19	18	13	16
Occasionally	40	26	24	18	22
Never	16	54	58	68	62
**Frequency of betting skins on external websites**					
Very often/fairly often ^(a: p<0.01; b: p<0.01)^	26	2	2	0	2
Occasionally	32	5	2	1	3
Never	42	93	96	98	95
**Frequency of betting skins privately**					
Very often/fairly often ^(a: p<0.01; b: p<0.01)^	32	2	2	0	2
Occasionally	25	7	4	2	4
Never	43	91	95	98	94
**Frequency of gambling**					
More than once a week ^(a: p<0.01; b: p<0.01)^	39	10	3	-	4
About once a week	27	12	5	-	4
About once a fortnight	5	9	5	-	3
About once a month	9	16	14	-	6
A few times a year	20	53	73	-	26
Not in the past 12 months	-	-	-	100	57
**Number of gambling activities undertaken**					
None	-	-	-	100	57
1	15	17	62	-	18
2	35	26	27	-	11
3	35	20	8	-	5
4	26	13	2	-	3
5 or more ^(a: p<0.00; b: p<0.05)^	50	24	1	-	5
**Problem Gambling Severity Index score**					
0: non gambler/non-problem gambling	20	51	69	100	82
1-2: low risk gambling	16	29	21	-	10
3-7: moderate risk gambling	11	11	6	-	4
8 or more: problem gambling ^(a: p<0.01; b: p<0.01)^	53	9	3	-	4
**Alcohol status**					
M-SASQ score 3+ ^(a: p<0.01; b: p=0.95)^	22	22	14	10	13
**Impulsivity**					
Mean impulsivity score ^(a: p<0.01; b: p<0.01)^	3.1	2.1	2.1	2.1	2.1

a,b Two significance tests were run: a) to see if the characteristic varied across all four categories (esports bettors to non-gamblers) and b) to see if there were differences between esports bettors and other sports bettors.

**Table 2:** Adjusted odds ratios for being an esports bettor.

	OR	95% CI (lower)	95% CI (higher)
**Sex (p = 0.14)**			
Male	1		
Female	0.65	0.37	1.16
**Age (p < 0.001)**			
16–18	1		
19–21	2.82	1.51	5.27
22–24	2.13	1.08	4.19
**Whether not in education, employment or training (p = 0.27)**			
**Yes**	0.61	0.25	1.47
**No**	1		
**Impulsivity (p = 0.11)**			
Impulse score	1.29	0.94	1.76
Frequency of playing digital games (p = 0.60)			
A few times a year/not at all	1		
About once a month	1.06	0.18	6.14
About once a fortnight	1.86	0.65	5.35
About once a week	1.85	0.70	4.93
More than once a week	1.33	0.52	3.38
**Frequency of paying money for loot boxes (p < 0.001)**			
Very often/fairly often	10.45	4.08	26.80
Occasionally	3.45	1.73	6.86
Never	1		
**Whether bought loot boxes using in-game items (p = 0.14)**			
Very often/fairly often	1.48	0.69	3.15
Occasionally	2.02	0.99	4.12
Never	1		
**Whether bet skins on external websites (p < 0.05)**			
Very often/fairly often	2.57	0.92	7.20
Occasionally	3.89	1.59	9.54
Never	1		
**Whether bet skins privately <p < 0.01)**			
Very often/fairly often	5.89	2.26	15.36
Occasionally	2.77	1.08	7.13
Never	1		

## Data Availability

Data from the Emerging Adults Gambling Survey will be made available via the UK Data Archive upon completion of the fellowship project. HW will consider reasonable requests in the interim.
